# Transdermal Delivery Systems for Ibuprofen and Ibuprofen Modified with Amino Acids Alkyl Esters Based on Bacterial Cellulose

**DOI:** 10.3390/ijms22126252

**Published:** 2021-06-10

**Authors:** Paula Ossowicz-Rupniewska, Rafał Rakoczy, Anna Nowak, Maciej Konopacki, Joanna Klebeko, Ewelina Świątek, Ewa Janus, Wiktoria Duchnik, Karolina Wenelska, Łukasz Kucharski, Adam Klimowicz

**Affiliations:** 1 Department of Chemical Organic Technology and Polymeric Materials, Faculty of Chemical Technology and Engineering, West Pomeranian University of Technology in Szczecin, Piastów Ave. 42, 71-065 Szczecin, Poland; joanna.klebeko@gmail.com (J.K.); ewelinaswiatek94@gmail.com (E.Ś.); ejanus@zut.edu.pl (E.J.); 2 Department of Chemical and Process Engineering, Faculty of Chemical Technology and Engineering, West Pomeranian University of Technology in Szczecin, Piastów Ave. 42, 71-065 Szczecin, Poland; rafal.rakoczy@zut.edu.pl (R.R.); maciej.konopacki@zut.edu.pl (M.K.); 3 Department of Cosmetic and Pharmaceutical Chemistry, Pomeranian Medical University in Szczecin, Powstańców Wielkopolskich Ave. 72, 70-111 Szczecin, Poland; anowak@pum.edu.pl (A.N.); wiktoria.duchnik@pum.edu.pl (W.D.); lukasz.kucharski@pum.edu.pl (Ł.K.); adam.klimowicz@pum.edu.pl (A.K.); 4 Department of Nanomaterials Physicochemistry, Faculty of Chemical Technology and Engineering, West Pomeranian University of Technology in Szczecin, Piastów Ave. 45, 70-311 Szczecin, Poland; karolina.wenelska@zut.edu.pl

**Keywords:** amino acid, bacterial cellulose, ibuprofen, non-steroidal anti-inflammatory drug, transdermal drug delivery, skin barrier

## Abstract

The potential of bacterial cellulose as a carrier for the transport of ibuprofen (a typical example of non-steroidal anti-inflammatory drugs) through the skin was investigated. Ibuprofen and its amino acid ester salts-loaded BC membranes were prepared through a simple methodology and characterized in terms of structure and morphology. Two salts of amino acid isopropyl esters were used in the research, namely L-valine isopropyl ester ibuprofenate ([ValOiPr][IBU]) and L-leucine isopropyl ester ibuprofenate ([LeuOiPr][IBU]). [LeuOiPr][IBU] is a new compound; therefore, it has been fully characterized and its identity confirmed. For all membranes obtained the surface morphology, tensile mechanical properties, active compound dissolution assays, and permeation and skin accumulation studies of API (active pharmaceutical ingredient) were determined. The obtained membranes were very homogeneous. In vitro diffusion studies with Franz cells were conducted using pig epidermal membranes, and showed that the incorporation of ibuprofen in BC membranes provided lower permeation rates to those obtained with amino acids ester salts of ibuprofen. This release profile together with the ease of application and the simple preparation and assembly of the drug-loaded membranes indicates the enormous potentialities of using BC membranes for transdermal application of ibuprofen in the form of amino acid ester salts.

## 1. Introduction

Transdermal drug delivery is one of the most important methods of delivering the drug to the body. Advantages associated with this include non-invasive delivery, bypass of first pass metabolism, prolonged duration of action of drugs [1]. One of the ways to deliver a transdermal anti-inflammatory drug is the application of various types of patches and membranes [2]. In recent years, attention has been paid to the use of natural materials, including bacterial cellulose (BC) membranes.

Cellulose is the most abundant biopolymer and biosynthesized by plants. Several bacteria can also produce an extracellular form of cellulose, commonly known as bacterial cellulose (BC). Similar to that of plant cellulose, BC shares the same molecular formula (C_6_H_10_O_5_)_n_. However, BC differs from conventional cellulose in its physical and chemical features. The two cellulose types bear the same chemical similarity being β-1,4-glucans, but differ in their degree of polymerization. The degree of polymerization for BC is considerably lower, having a typical polymerization range between 2000 and 6000 compared to 13,000–140,000 of plant cellulose [3,4].

The BC is produced in the form of highly swollen membranes with high water content (>90%) [2]. The BC has good physical and mechanical properties, high purity, and biocompatibility, which engendered considerable interest in this material in the biomedical field [2,5,6], among others specifically as wound healing membranes [5,7] scaffolds for tissue engineering [8,9], artificial blood vessels [3,5].

One of the proposed applications is transdermal drug delivery; the evidence shows that BC has good skin tolerance and does not cause irritation [10]. BC has been used in several systems for delivery such drugs as ibuprofen, lidocaine [11] diclofenac, caffeine [2], silver sulfadiazine [12] as well as amoxicillin [13].

A popular and frequently used transdermal drug to relieve skin inflammation and subcutaneous tissue is ibuprofen (IBU) [14]. IBU is a non-steroidal anti-inflammatory drug (NSAIDs), commonly used to relieve pain and inflammation [1]. IBU is a well-tolerated compound with antipyretic and pain relief properties. IBU acts through the inhibition of two isoforms of cyclooxygenase (COX-1 and COX-2), enzymes that are responsible for prostaglandin biosynthesis [15]. Although NSAIDs, including IBU, are very effective, their oral absorption could be associated with severe gastric irritation leading to gastric bleeding and ulcers. Therefore, transdermal delivery is preferred as it bypasses hepatic first pass metabolism [1]. The use of BC as a drug delivery system is very interesting and had already been explored for ibuprofen [11] and some ibuprofen salts derivatives (namely, ionic liquids derivatives) [16].

This study aimed to investigate the potential of BC membranes as systems for transdermal drug delivery for new derivatives of ibuprofen. It is known from our previous publication that the ionic pairs of ibuprofen with the biocompatible counter-ions of L-valine alkyl ester have better penetration through porcine skin compared to the starting acid—ibuprofen. At the same time, it was shown that the best results were obtained for the isopropyl ester-[ValOiPr][IBU] (13.55-fold higher than the parent drug); therefore, this compound was used in this study [17]. The results were compared for the new L-leucine isopropyl ester salt and the parent drug—ibuprofen. It is worth emphasizing that the use of amino acid alkyl ester salts will not change the effectiveness of the drugs themselves, because in the body these compounds will be converted into the known form, ibuprofen, due to hydrolysis. However, it will increase the speed of their delivery to the body easily and safely.

## 2. Results and Discussion

### 2.1. Identification and Properties of [LeuOiPr][IBU]

The synthesis of amino acid isopropyl ester of ibuprofen was performed using the previously described three-step method [17]. The synthesis and properties of the L-valine isopropyl ester salt ([ValOiPr][IBU]) have been described in previous publications [17,18]. This study publication uses a new salt-the L-leucine isopropyl ester salt ([LeuOiPr][IBU]); therefore, only the results are presented for this compound.

[LeuOiPr][IBU]—L-leucine isopropyl ester ibuprofenate.


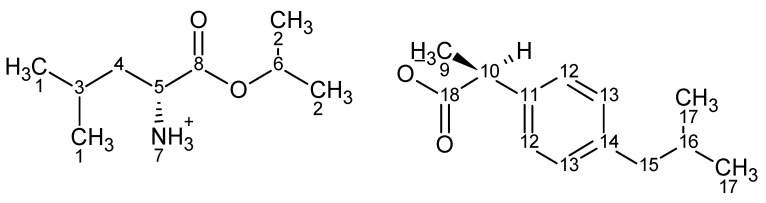


L-leucine isopropyl ester ibuprofenate was obtained as a white solid in 94% yield and was identified by ^1^H and ^13^C NMR, FTIR, and elemental analysis.

^1^H NMR (400 MHz, CDCl_3_) δ in ppm: 7.21 (d, 2H, J12,13 = 8.0 Hz, H12); 7.07 (d, 2H, J13,12 = 8.0 Hz, H13); 5.95 (s, 3H, H7); 4.99–5.05 (m, 1H, H6); 3.62–3.64 (q, 1H, H10); 3.48–3.52 (q, 1H, H5); 2.43 (d, 2H, J15,16 = 7.1 Hz, H15); 1.82–1.85 (m, 1H, H16); 1.71 (t, 1H, H4); 1.51–1.58 (m, 1H, H3); 1.40–1.47 (d, 3H, H9; t, 1H, H4); 1.22 (dd, 6H, H2); 0.86–0.90 (d, 6H, H1, d, 6H, H17); 13C NMR (100 MHz, CDCl3) δ in ppm: 179.17 (C18); 173.34 (C8); 140.12 (C11); 138.45 (C14); 129.12 (C12); 127.19 (C13); 68.71 (C6); 52.08 (C5); 45.66 (C15); 45.00 (C10); 42.91 (C4); 30.13 (C16); 24.56 (C3); 22.67 (C17); 22.35 (C1); 21.82 (C1); 21.67 (C2); 21.63 (C2); 18.48 (C17); FT-IR: ν(ATR): 2980; 2971; 2931; 2904; 2891; 2870; 1739; 1614; 1562; 1530; 1514; 1463; 1386; 1358; 1366; 1327; 1287; 1225; 1189; 1179; 1164; 1101; 1061; 951; 908; 876; 861; 813; 798; 729; 546 cm−1; elemental analysis: calc. (%) for C22H37NO4 (379.54): C 70.60, H 9.86, N 3.70, O 16.86, found: C 70.59, H 9.85, N 3.69, O 16.86; UV-Vis (EtOH): λ_max_ = 229.0 nm; T_m_ = 86.7 °C; [α]_D_^20^ = +9.162 (c = 1.021 g/100 mL EtOH).

^1^H and ^13^C NMR spectra, FTIR spectra, and TG curves for [LeuOiPr][IBU] are available in Figures S1–S4 (Supplementary Materials).

The identity and purity of the obtained compound were confirmed. In addition, its properties were compared to the previously obtained L-valine ester [17]. It has been shown that the elongation of the alkyl chain in the amino acid molecule influences the stability, lipophilicity, and melting point of the obtained compound. [ValOiPr][IBU] has a lower melting point (T_m_ = 78.01 °C) compared to [LeuOiPr][IBU] (T_m_ = 86.7 °C). The thermal stability increased with the length of the alkyl chain. The ion pair of ibuprofen and l-valine isopropyl ester [LeuOiPr][IBU] had higher thermal stability than [ValOiPr][IBU] and amounted to 108.1 °C and 90.2 °C, respectively. It was also confirmed that the longer the alkyl chain, the higher lipophilicity of the compound. The n-octanol/water partition coefficients were 1.40 for [LeuOiPr][IBU] and 1.15 for [ValOiPr][IBU], respectively.

[LeuOiPr][IBU] has a chiral center since it was obtained from optically pure L-leucine. The ibuprofen used in the syntheses was a racemic mixture. The value of specific rotation +9.162 (molar optical rotation: +34.773) for [LeuOiPr][IBU] was lower compared to the +11.852 (molar specific rotation: +43.325) for [ValOiPr][IBU].

### 2.2. Preparation and Characterization of BC, BC-IBU, BC-[ValOiPr][IBU], and BC-[LeuOiPr][IBU] Membranes

**Figure 1:**
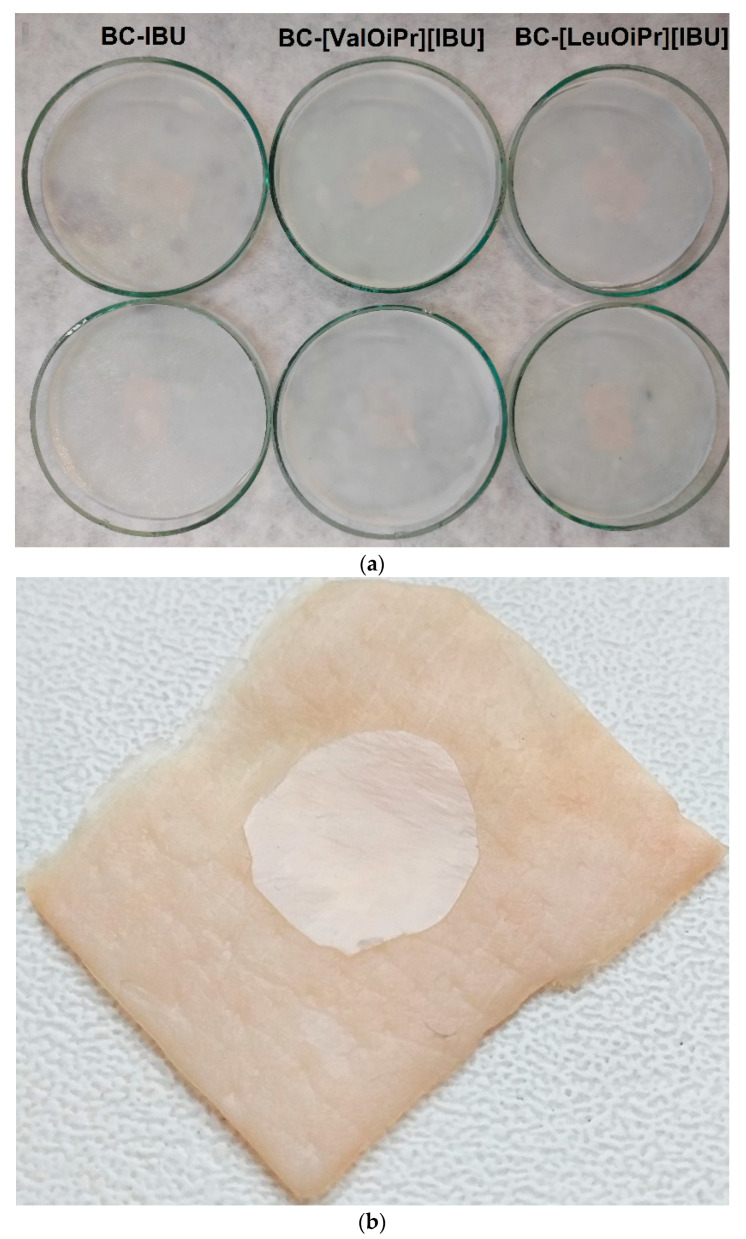
(**a**) Images of BC-IBU, BC-[ValOiPr][IBU] and BC-[LeuOiPr][IBU] dry membranes and (**b**) one example (BC-[LeuOiPr][IBU] membrane) showing good dermal adherence.

BC-IBU, BC-[ValOiPr][IBU] and BC-[LeuOiPr][IBU] membranes were prepared by complete incorporation of an established volume of ibuprofen or its amino acid isopropyl ester salts solutions into drained BC-membranes. The objective of preparing BC-ibuprofen membranes with these three compounds was to compare how the modification of the compound will affect its properties and applicability in preparations applied to the skin. This method made it possible to obtain BC membranes modified with ibuprofen or its salts simply and quickly. Using this method, the exact mass of ibuprofen introduced into the membrane was determined, which was additionally confirmed by weighting the dry BC membranes and by measuring, by HPLC, the total amount of drug released in the dissolution assays. The obtained membranes were homogeneous, as shown in Figure 1a, indicating a good dispersion of ibuprofen or its modification inside the tridimensional nanofibrillar network of BC, without the formation of aggregates.

**Figure 2:**
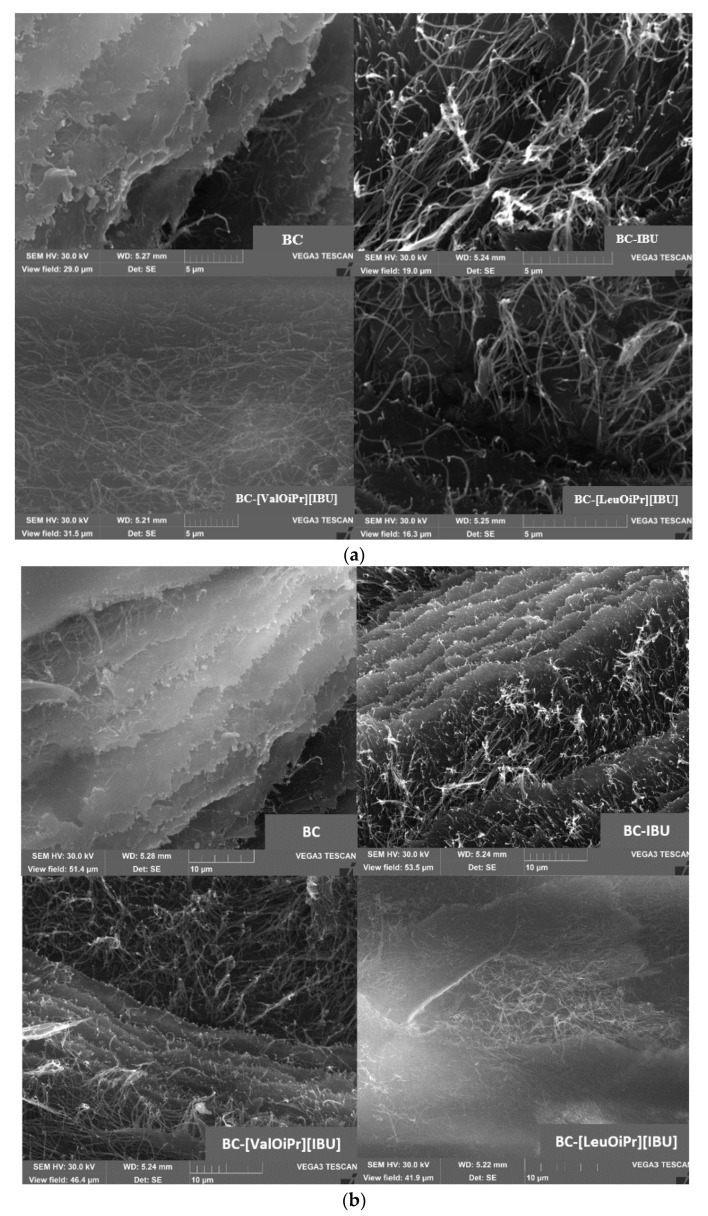
SEM images of BC, BC-IBU, BC-[ValOiPr][IBU] and BC-[LeuOiPr][IBU] (**a**) Scale of 5 μm (**b**) Scale of 10 μm.

The uniformity of the BC membranes was further confirmed by surface SEM analysis (Figure 2). The SEM images disclosed also, a good dispersion of ibuprofen and ibuprofen derivatives in the BC membranes surface, as no formation of considerable aggregates or crystallized drug is perceivable. In pure BC membranes, fewer spaces between the nanofibrils were observed because of the collapse of the structure upon drying, while in the BC-ibuprofenate membranes these spaces were wider, showing a more open structure, because of the presence of API. Ibuprofen or ibuprofen amino acid isopropyl ester salts will be retained inside the BC network, reducing the attraction forces between the nanofibrils, maintaining some mobility of the nanofibres, and therefore, avoiding their collapse during drying and keeping some flexibility. At the same time, it can be seen in Figure 2b that the cellulose has retained its original structure.

**Figure 3:**
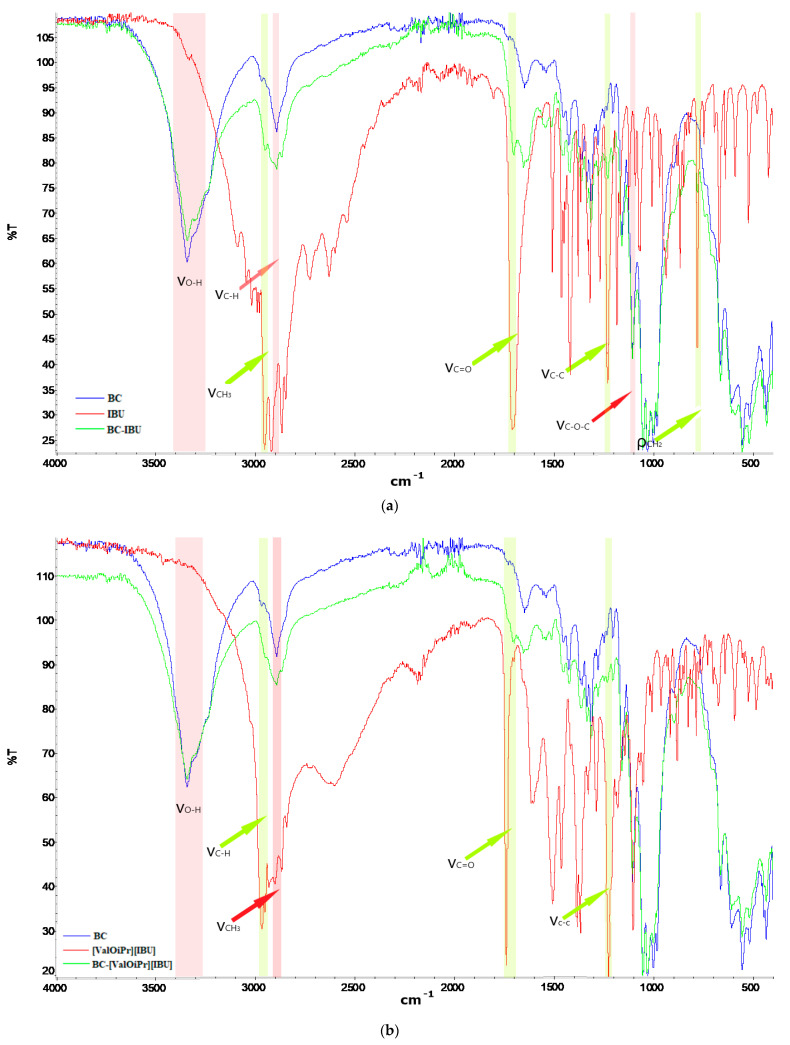

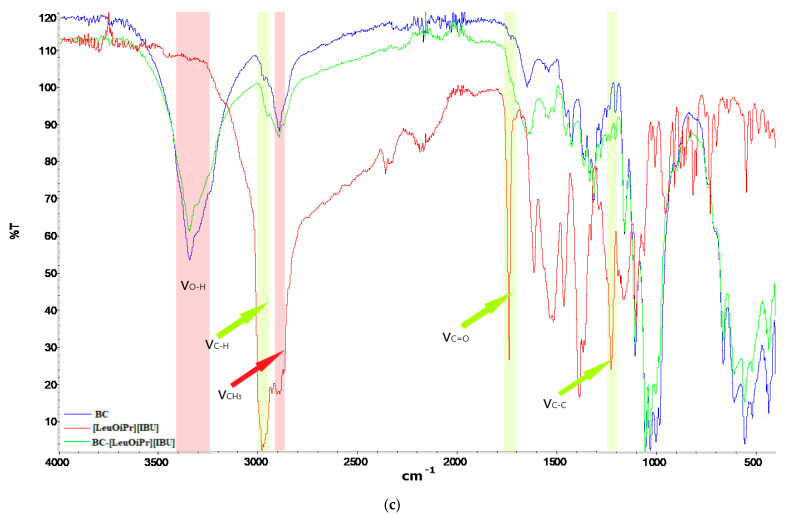
FTIR-ATR spectra of bacterial cellulose and API (**a**) BC (blue), IBU (red) and BC-IBU (green), (**b**) BC (blue), [ValOiPr][IBU] (red), and BC-[ValOiPr][IBU] (green), (**c**) BC (blue), [LeuOiPr][IBU] (red), and BC-[LeuOiPr][IBU] (green).

The structural characterization of all obtained membranes was carried out by ATR-FTIR. The spectra of BC, BC-IBU, BC-[ValOiPr][IBU], and [LeuOiPr][IBU] are presented in Figure 3. Pure BC membrane showed the typical FTIR spectrum of cellulosic materials, with the main absorption bands at 3343, 2894, and 1160 cm^−1^, attributed to the vibrations of the O-H, C-H, and C-O-C groups, respectively. The very strong band at 2954 cm^–1^ in the FTIR spectrum of ibuprofen is assigned to CH_3_ asymmetric stretching [19,20,21]. Ibuprofen has also shown the presence of free acid carbonyl peak at 1709 cm^–1^ with high intensity, and a very high-intensity peak of ibuprofen at 1230 cm^–1^ was due to C-C stretching. A strong band noticed at 779 cm^–1^ in ibuprofen was due to CH_2_ rocking vibration. Moreover, there were typical absorption peaks at 1573 cm^−1^ (COO anti-symmetrical vibration) and 1603 cm^−1^ (C-C ring skeletal vibration) [22]. In the [ValOiPr][IBU] and [LeuOiPr][IBU] spectra, there are additional absorption bands characteristic of vibrations in the functional groups derived from the amino acid, in particular at 1383 and 1386 cm^−1^ (C-N stretching), respectively [17]. The BC-IBU, BC-[ValOiPr][IBU] and BC-[LeuOiPr][IBU] spectra are essentially a sum of the vibration peaks of all individual components, with no appearance of novel peaks or shifts in peaks position, indicating the nonattendance of complex interactions between BC and pharmaceutical active ingredients. Interestingly, in the ATR-FTIR spectra of drugs-loaded BC membranes, the characteristic peak at 1709 cm^−1^ is not visible, which can testify that the active compound is more incorporated inside the BC membrane than at the surface. Moreover, FTIR spectra collected at different points of the surface and inner layers of the ibuprofen or its amino acid isopropyl ester salts loaded membranes showed a similar profile, confirming the good dispersion of API inside the membranes.

The flexibility of the obtained membranes guaranteed satisfactory adherence to the skin, which is shown in Figure 1b. The mechanical properties of the obtained membranes such as Young’s modulus, tensile strength, and elongation at break were determined and listed in Table 1. It was noticed that the modification of BC with ibuprofen and its salts did not significantly change the mechanical properties of the membranes. It was observed that the modification of the membrane slightly increased the tensile strength as well as elongation at break. It also increased Young’s modulus, which may indicate a slight stiffening of the membranes.

### 2.3. Dissolution Assays

**Figure 4:**
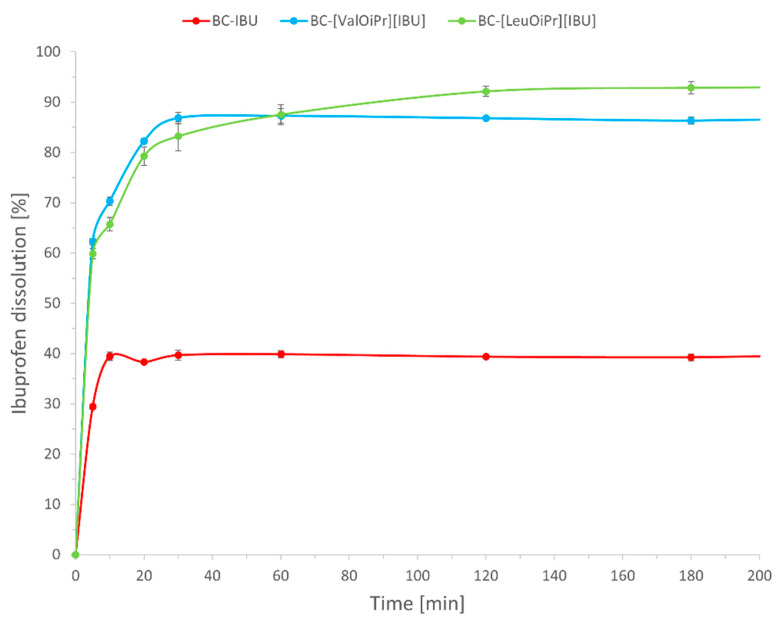
Dissolution profiles of BC-IBU, BC-[ValOiPr][IBU] and BC-[LeuOiPr][IBU]. Mean values ± standard deviation, *n* = 3.

The release profile of active ibuprofen from BC-IBU, BC-[ValOiPr][IBU], and BC-[LeuOiPr][IBU] membranes in a phosphate buffer solution are displayed in Figure 4. About 30% from BC-IBU and about 60% from BC-[ValOiPr][IBU] and BC-[LeuOiPr][IBU] of the total drug was released in the first five minutes. The release profile is slightly different depending on the compound used to modify the bacterial cellulose. Therefore, for BC-IBU the maximum release at the level of 40% was achieved after about 10 min, for BC-[ValOiPr][IBU] about 87% after 30 min, and BC-[LeuOiPr][IBU] over 90% after 120 min. Modification of ibuprofen resulted in a more than twofold increase in dissolution. Lower values for BC-IBU are associated with lower solubility of ibuprofen compared to its amino acid ester salts. Amino acid isopropyl ester ibuprofenates are water-soluble, its release from the BC membranes is essentially governed by its diffusion through the porous tridimensional network of BC. It was observed that the smaller molecule, non-modified ibuprofen, diffuses faster and reaches the maximum concentration faster. Previous studies with BC-diclofenac and BC-lidocaine membranes showed very similar dissolution profiles [2,11].

### 2.4. Permeation Studies

The resulting combination of ibuprofen with amino acid isopropyl esters proved to be an effective and simple modification of ibuprofen that increased its permeability through the skin. In this article, we compare the permeability of ibuprofen and its amino acid ester salts from BC membranes.

In recent years, BC has considerable interest in several fields, but particularly in the biomedical area, for example, in wound dressings as well as of controlled drug delivery systems [10,23,24]. Bacterial cellulose (BC) is a promising material for wound healing due to its outstanding properties of holding water, strength, and degradability [12]. Recent studies indicated, that BC can be successfully applied to modulate the bioavailability of IBU [11].

In our study, it has been investigated the applicability of BC membrane in transdermal drug delivery systems, wherein the permeation of two ibuprofen derivatives ([ValOiPr][IBU] and [LeuOiPr][IBU]), was compared with the permeation of the free acid (IBU). Permeation through porcine skin at 37 °C was studied using the Franz diffusion cell. The porcine skin is frequently used for preliminary evaluation of percutaneous permeation of transdermally applied drugs. Numerous histopathological studies confirmed its similarity to human skin [25,26].

**Figure 5:**
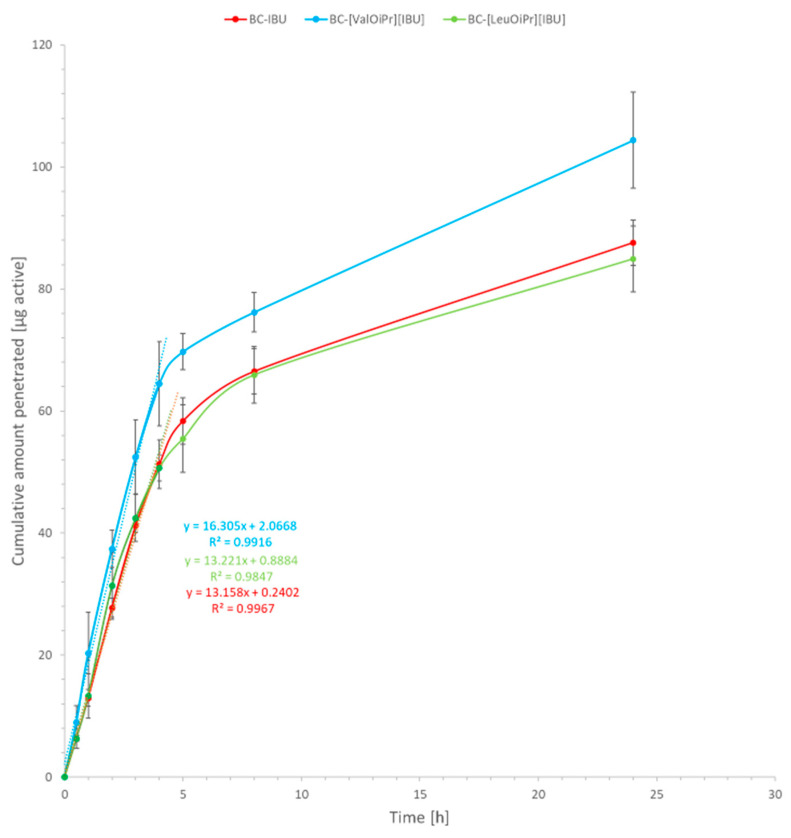
Ibuprofen permeation across pigskin. Mean values ± standard deviation, *n* = 3.

**Figure 6:**
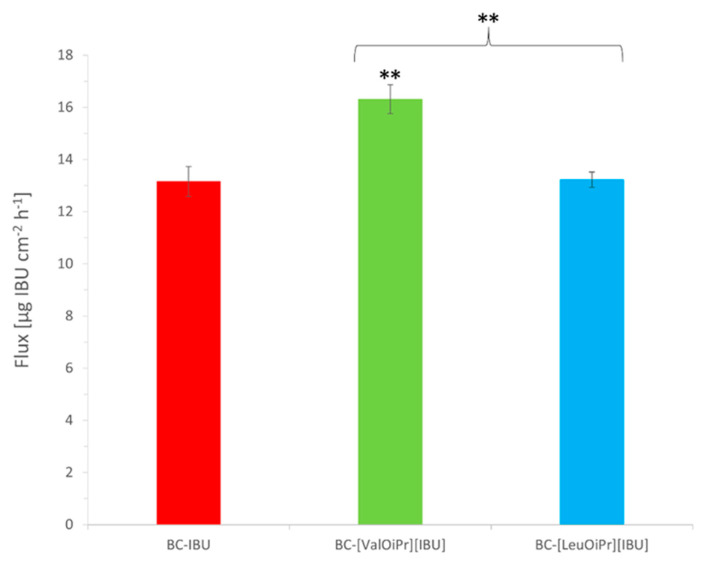
Flux values for ibuprofen permeation from BC membranes. Mean values ± standard deviation, *n* = 3. Statistically significant difference from BC-IBU was estimated using the ANOVA test. Double asterisk ** means statistical difference for *p* < 0.01. Brace { means statistical difference between two ibuprofenate salts.

Degree of permeation defined as a cumulative mass after the 24 h experiment is shown in Table 2. The permeation profiles of ibuprofen from BC-IBU, BC-[VaOiPr][IBU], and BC-[LeuOiPr][IBU] membranes through pigskin are shown in Figure 5 and Figure 6.

The profiles of ibuprofen permeation are very useful to obtain the permeation parameters such as the steady-state permeation flux, the diffusion coefficient, and the time required to reach steady-state permeation (lag time). The steady-state fluxes (J_SS_) of ibuprofen and its derivatives through the skin were calculated from the slope of the plot of cumulative mass in the acceptor phase over time and were expressed as the amount of active ibuprofen per skin area and time (μg IBU cm^–2^ h^–1^). Depending on the type of modification in API, different steady-state flux values (Jss) were obtained; the highest ibuprofen fluxes were attained with BC-[ValOiPr][IBU] (16.31 ± 0.55 μg IBU cm^–2^ h^–1^) and the lowest with the BC-IBU (13.16 ± 0.57 μg IBU cm^–2^ h^–1^) membrane. All other obtained membranes showed similar BC-IBU flux values, with no significant differences among them. A comparison of the different ibuprofen derivatives used in BC membranes based on the permeability coefficient K_p_ provided the same trends observed in the fluxes (Table 3). Lag time (L_T_) was determined by extrapolating the equation, and as can be seen, it depended on the size of the API molecule used in BC.

Compared to the results from our previous study [18] on the permeation of amino acid alkyl esters ibuprofenates from alcohol solutions, the results of steady-state permeation flux, the diffusion coefficient, and the time required to reach steady-state permeation, were slightly lower than the results obtained with bacterial cellulose membranes.

The results of ibuprofen permeability from bacterial cellulose presented in this paper are slightly higher than those presented by Trovatti and co. and were, respectively, 9.86 ± 0.25 and 6.83 ± 0.64% applied dose after 8 h of permeation. The cellulose used in previous studies was produced using the bacteria *Gluconacetobacter sacchari* [11].

All tested compounds permeated the skin, wherein [ValOiPr][IBU] had a significantly higher degree of permeation of IBU. After a 24-h permeation experiment, the highest average cumulative mass (statistically significant) was for [ValOiPr][IBU] (104.402 ± 7.875), while for IBU—87.614 ± 3.737 and similar to the [LeuOiPr][IBU] (84.944 ± 5.394) (Table 2). The permeation of [ValOiPr][IBU] derivatives was significantly higher compared to IBU, which was confirmed also the by Wilcoxon test (*p* = 0.0500) (Table S2) and by the cluster analysis test (Figure S5). In Table 2, BC-[LeuOiPr][IBU] presents lower cumulative mass permeated and lower skin accumulation, comparing to BC-IBU with the suggestion that more quantity of [LeuOiPr][IBU] stays inside the membrane than BC-IBU.

In other studies, the BC was assessed for IBU permeation through the human epidermis in the Franz diffusion cell [11]. These authors showed permeation rate was almost threefold higher for IBU-loaded BC than for IBU located in gel and PEG400 solution [11]. In other studies, using human skin, in vitro permeation showed that diclofenac sodium loaded BC had a permeation rate comparable to marketed patches containing diclofenac sodium and significantly lower than of a commercial gel formulation [10], whereas the BC containing carboxymethyl cellulose and epichlorohydrin (cross-linker) were analyzed for controlled drug delivery using ibuprofen sodium (IbuNa). This study showed, that the carboxymethyl cellulose content and epichlorohydrin concentration influenced the swelling and drug release properties of the hydrogels [13].

In permeation testing, it is important to determine the permeation efficiency. For some drugs, such as NSAIDs, faster permeation is preferable to achieve a rapid therapeutic effect. Increased permeation in less time causes a faster decrease in inflammation. It is more desirable than advisable to incorporate drugs with increased permeation into transdermal drug delivery.

**Figure 7:**
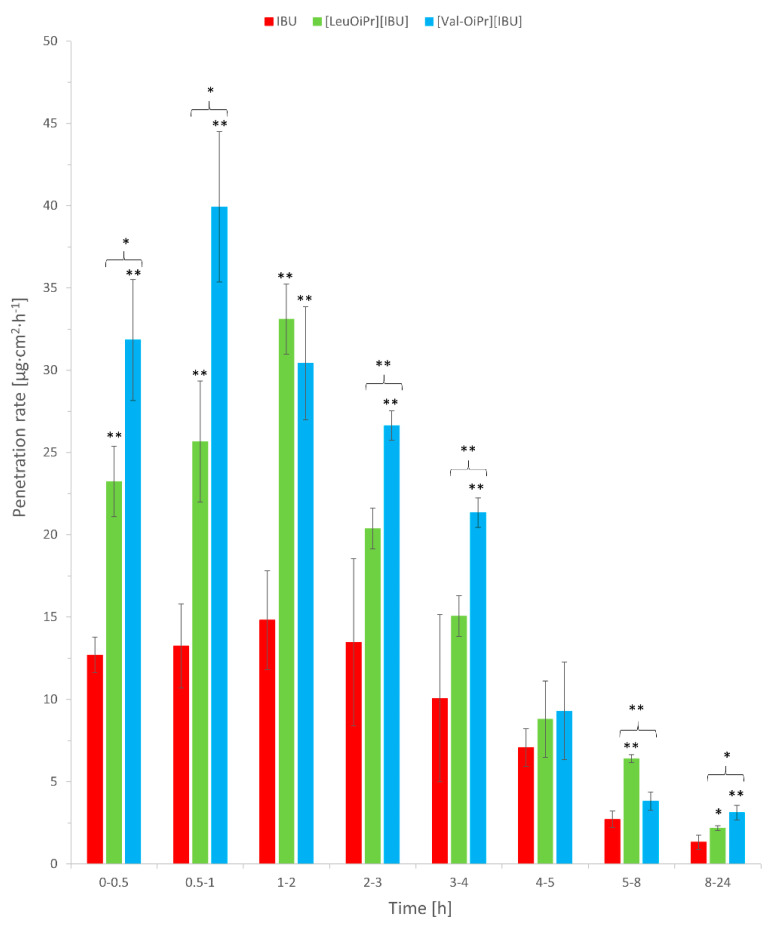
The permeation rate of IBU, [ValOiPr][IBU], and [LeuOiPr][IBU], permeated from BC into acceptor fluid. Mean values ± standard deviation, *n* = 3. Statistically significant difference from IBU was estimated using the ANOVA test. Double asterisk ** means statistical difference for *p* < 0.01, single asterisk * means statistical difference for *p* < 0.05. Brace { means statistical difference between two ibuprofenate salts.

In our study, the rate permeation of IBU derivatives and acid IBU from BC to acceptor liquid is showed in Figure 7. IBU derivatives permeated from BC fastest in the first hours of the study. The sudden increase in permeation was observed in 0.5–1 time point (0.5–1 h) for [ValOiPr][IBU] and in 1–2 time point (1–2 h) for [LeuOiPr][IBU]. In contrast, IBU permeated at a constant level of 4–5 h (Figure 7), which would mean that the introduced modification of ibuprofen would allow obtaining a faster therapeutic effect.

The delivery of ibuprofen through the skin takes place by passive diffusion [27]. In the case of IBU derivatives, the effect of penetration increasing the of the active substance is associated with the formation of ion pairs that easily penetrate the lipids and dissociate in the living layers of the skin [28,29,30]. Moreover, the increase in penetration may be obtained as a result of a change in the solubility of the drug in the donor phase [31].

Cellulose fibers have a slight negative (anionic) charge. The ionizing (acidic) groups on cellulose originate from cell wall constituents [32]. The large surface area and negative surface charge at BC make it an excellent drug carrier for hydrophilic drugs. Therefore, drugs can attach to the BC surface with optimal control of the dose. Thus, a reduction in the lipophilicity of ibuprofen (logP = 2.415 ± 0.001) by the use of an amino acid ester derivative should therefore be advantageous for use in transdermal patches using bacterial cellulose. The compounds used in the research were characterized by higher hydrophilicity and their partition coefficient was for [ValOiPr][IBU] and [LeuOiP][IBU] logP = 1.154 ± 0.004 and 1.404 ± 0.003, respectively. [ValOiPr][IBU] has been shown to have increased skin permeability. Considering the release profile of BC, the permeability, and solubility of this compound, it can be concluded that this compound shows high potential as an alternative to the use of ibuprofen. The obtained results for [LeuOiPr][IBU] suggest that the higher lipophilicity, molecular weight, and alkyl chain length of the leucine derivative compared to the valine derivative adversely affects the skin permeability. The leucine derivative shows similar results to ibuprofen alone despite lower lipophilicity and higher solubility.

Table 2 showed the number of tested compounds accumulated in the porcine skin, determined in 24 h of permeation expressed in µg IBU/g of skin. The highest accumulation in the skin was for IBU (293.556 ± 53.197), next [LeuOiPr][IBU] (119.247 ± 13.608), and [ValOiPr][IBU] (106.516 ± 16.338)—Table 2, Figure S6.

The applied compounds may both penetrate and accumulate in the skin. In our study, it was observed that IBU derivatives accumulate in the skin in a lower amount compared to IBU, which was caused by the greater permeation of the studied derivatives.

## 3. Materials and Methods

### 3.1. Materials

All reagents were commercially available materials and were used without further purification. (RS)-Ibuprofen (99%) was obtained from Acros Organics (Geel, Belgium). L-valine and L-leucine (≥99%) were purchased from Carl Roth (Karlsruhe, Germany). Trimethylsilyl chloride (≥99%) (TMSCl) was provided by Sigma-Aldrich (Steinheim am Albuch, Germany). Methanol, propan-2-ol (iPrOH), potassium chloride, sodium chloride, orthophosphoric acid (98%), pH = 4 buffer, citric acid, sodium hydroxide were high purity obtained from Chempur (Piekary Ślaskie, Poland). Ammonium hydroxide solution 25% (NH_3_∙H_2_O) was of analytical grade purchased from StanLab (Lublin, Poland). Acetonitrile (≥99.9%) for HPLC gradient grade was provided by Sigma-Aldrich (Steinheim am Albuch, Germany). Anhydrous disodium hydrogen phosphate (≥99%) (Na_2_HPO_4_), anhydrous potassium dihydrogen phosphate (99%) (KH_2_PO_4_) were provided by Merck (Darmstadt, Germany). Yeast extract and bacto-peptone were obtained from Graso BIOTECH (Starogard Gdański, Poland). D-mannitol (≥99.9%) was provided by POL-AURA (Dywity, Poland).

### 3.2. Synthesis of the Bacterial Cellulose

Bacterial cellulose (BC) was produced employing *Komagataeibacter xylinus* bacteria strain (ATCC^®^ 53524™). Modified buffered S&H 1717 ATCC medium was used for bacteria cultivation and BC production. The medium base consists of bacto-peptone 5 g/L, yeast extract 5 g/L, Na_2_HPO_4_ 2.7 g/L, citric acid monohydrate 1.15 g/L. The medium base was dissolved in distilled water and autoclaved at 121 °C for 15 min. Then, the carbon-source aqueous solution filter-sterilized was aseptically added to the medium to reach the concentration of 20 g/L. Finally, the bacteria medium was buffered to reach pH = 5. Bacteria inoculum was prepared to transfer 100 µL of a liquid aliquot of the original stock sample to a 15 mL plastic tube and then cultivating for 7 days at 30 °C.

For BC production a 1.2 L of cultivation medium was placed in a plastic litter box (inner dimensions: 253 × 325 × 57 mm) and a content of a single inoculum tube was transferred into the medium. Then, the litter box was secured by a food wrap and placed for 8 days in the incubator at 30 °C. After incubation, harvested BC was three times washed with distilled water to remove the medium remains and immersed in 0.1 M NaOH solution at 80 °C for 30 min to remove bacteria cells. Then, BC was again washed by the distilled water to remove the NaOH residuals, until pH = 7 was reached.

### 3.3. Preparation of Bacterial Cellulose (BC), Bacterial Cellulose Loaded Ibuprofen (BC-IBU), Bacterial Cellulose Loaded L-Valine Isopropyl Ester Ibuprofenate (BC-[ValOiPr][IBU]), and Bacterial Cellulose Loaded L-Leucine Isopropyl Ester Ibuprofenate (BC-[LeuOiPr][IBU])-Membranes

Wet 140 mm × 8 mm circular BC membranes were weighted and handily compressed to remove 50–60% of their water content. Drained BC membranes were then soaked in a solution of ibuprofen or a salt thereof dissolved in the mixture of 3 mL of aqueous buffered solution (pH 7.4) and 2 mL of ethanol, for 24 h at room temperature to assure complete absorption of the drug. After the total absorption of the solution, the BC membranes were dried at 50 °C in a ventilated oven for 12 h. BC membranes were prepared according to this method without adding API (active pharmaceutical ingredient). All membranes were kept in a desiccator until their use. The identification of all samples prepared is summarized in Table S1 (see Supplementary Materials). The content based on active ingredients was always the same in the membrane and was about 50 mg IBU per 1 g membrane. A fixed dose of the active substance was used in the studies. A limitation in the amount of drug loaded is its solubility in the solvent used.

### 3.4. General Analytical Method

#### 3.4.1. Identification and Properties of L-Leucine Isopropyl Ester Ibuprofenate ([LeuOiPr][IBU])

The NMR spectra were recorded with BRUKER DPX-400 spectrometer (Billerica, MA, USA) at 400 MHz (^1^H) and 100 MHz (^13^C) in CDCl_3_ as a solvent. The chemical shifts (δ, ppm) are given relative to TMS used as the internal standard.

The FTIR data were collected on Thermo Scientific Nicolet 380 spectrometer (Waltham, MA, USA) equipped with an ATR diamond plate. The spectra were recorded in transmission mode in the range 400–4000 cm^−1^ at the resolution of 4 cm^−1^.

UV-Vis spectra were recorded on Spectroquant^®^ Pharo 300 Spectrophotometer from Merck (Darmstadt, Germany). The solutions were prepared in absolute ethanol of concentration range 10^−4^–10^−5^ M. The measurements were performed in 10 mm quartz cell in the wavelength range 190–400 nm with the accuracy of ±1 nm.

The determination of elemental composition was performed using Thermo Scientific™ FLASH 2000 CHNS/O Analyzer (Waltham, MA, USA). The individual elements were detected by a thermal conductivity detector (TCD). The reactor temperature is 1060 °C for oxygen analysis, 950 °C for the rest of the elements. Three replications were performed for each compound. The samples were prepared in the tin (CHNS analysis) or silver (O analysis) crucibles and were weighed with an accuracy of ±0.000001 g. The content of individual elements was determined using the calibration curve method. 2,5-(Bis(5-tert-butyl-2-benzo-oxazol-2-yl)thiophene, L-cysteine, L-methionine, and sulphanilamide were used as standards in CHNS-mode, and acetanilide and benzoic acid were used for calibration in O-mode, respectively.

The thermal stability of the compounds was tested on a Netzsch Proteus Thermal Analysis TG 209 F1 Libra apparatus (Selb, Germany). The analysis was performed under an oxidizing atmosphere, nitrogen flow was 10 mL/min and nitrogen flow was 25 mL/min. The temperature range was 25–1000 °C. The tests were carried out with the use of alumina crucibles (Al_2_O_3_), and the sample weight was about 5 mg.

The specific rotation was tested using the Autopol IV automatic polarimeter from Rudolph Research Analytical (Hackettstown, NJ, USA) at the temperature of 20 °C and in 589 nm the wavelength.

The n-octanol/water partition coefficient was investigated by the shake flask method. The concentration of the substance in the aqueous layer was determined by high-performance liquid chromatography HPLC with a DAD (diode-array detection)/ FLD (fluorescence detector) detector. The SHIMADZU Nexera-i LC-2040C 3D High Plus liquid chromatograph (Kioto, Japan) was used for the tests. A mixture of 50% acetonitrile and 50% of water was used as the mobile phase. The flow rate was 1 mL/min. The column temperature was 30 °C. A Kinetex^®^ 2.6 µm F5 100 Å column with dimensions 150 × 4.6 mm^2^ was used from Phenomenex (Torrance, CA, USA). The injection volume for these samples was 50 µL. Each measurement was performed in triplicate and the results were averaged.

Liquid chromatography system (Knauer, Berlin, Germany) in skin permeation experiments for determination of compounds concentration in acceptor fluid and accumulation in the skin consisted of the following units: Smartline model 1050 pump, model 2600 UV detector, Smartline model 3950 autosampler model, model ClarityChrom 2009 integrator. The chromatographic column 125 × 4 mm column filled with 5 µm Hyperisil ODS (Thermo Scientific, Waltham, MA, USA) was applied (C9). The detection wavelength was 220 nm. The mobile phase of 0.02 M potassium dihydrogen phosphate-acetonitrile-methanol (53/40/7, *v*/*v*/*v*) adjusted to pH 2.5 with orthophosphoric acid was pumped at a flow rate of 1 mL/min. The column temperature was set at 25 °C, and the injection volume was 20 µL.

#### 3.4.2. Characterization of BC, BC-IBU, BC-[ValOiPr][IBU], and BC-[LeuOiPr][IBU]-Membranes

BC and BC-ibuprofen, BC-[ValOiPr][IBU] and BC-[LeuOiPr][IBU] dried membranes were characterized in terms of structure, surface morphology, and tensile mechanical properties.

Fourier transform infrared (FTIR) spectra were obtained in a Thermo Scientific Nicolet 380 spectrometer (Waltham, MA, USA) equipped with an ATR diamond plate. Thirty-two scans were acquired in the 400–4000 cm^−1^ range with are a resolution of 4 cm^−1^.

SEM micrographs of all obtained BC membrane surfaces were obtained on Tescan Vega 3 (Brno, Czech Republic), operating at 30 kV. Two samples of each membrane were analyzed.

Tensile assays were performed on an Instron 5982 testing machine (Norwood, MA, USA) in tensile mode with a 1 kN load cell. The test is conducted at tensile rates 10 mm/min until the specimen fails (yields or breaks). The samples were strips of 10 mm × 0.3 mm and the gauge length 70 mm. At least 5 specimens were tested from each sample. The corresponding stress (MPa)–strain (%) curves were plotted, and Young’s modulus was determined.

### 3.5. In Vitro Ibuprofen Release

#### 3.5.1. Dissolution Assays

BC-ibuprofen, BC-[ValOiPr][IBU] and BC-[LeuOiPr][IBU] dried membranes were immersed in a vessel containing 50 mL of a 0.01 M phosphate buffer (pH 7.4) solution. The dissolution was then carried out at 32 °C and 50 rpm. At determined time intervals, 0.5 mL of each solution was withdrawn, and the same volume of fresh buffer solution was added to maintain a constant volume. The active ibuprofen content in each aliquot was determined by the HPLC method. The method is the same as for lipophilicity testing.

#### 3.5.2. Permeation and Skin Accumulation Studies

In the experiment, porcine skin was used because it has similar permeability to human skin [33]. Numerous histopathological studies confirmed its similarity to human skin [25,26]. The skin used for the tests came from a pig’s abdomen and was delivered from a local slaughterhouse. The skin contained single hair, while selected for experiment fragments 1 cm^2^ in diameter, which were devoid of hairThe fresh abdominal porcine skin was washed in PBS buffer pH 7.4 several times. The pigskin with 0.5 mm thickness was excision by dermatomed [23,24,34], wrapped into aluminum foil, and stored at −20 °C until use. Under this condition, the skin was used no later than 3 months. This freezing time ensures the stability of the skin barrier properties [35].

Before the examination, the skin was thawed at room temperature for about 30 min, and then it was soaked in a PBS solution for 15 min to hydrate it [34,36]. In the next stage, the skin was mounted in Franz diffusion cells. The undamaged skin pieces with an even thickness were chosen for experiments. The undamaged skin pieces were placed between the donor and acceptor chamber of Franz diffusion cells, then their integrity has been checked by checking its impedance.

Skin impedance was measured using an LCR meter 4080 (Conrad electronic, Germany), which was operated in parallel mode at an alternating frequency of 120 Hz (error at kΩ values < 0.5%). For the measure of skin impedance, a donor chamber with a capacity of 2 mL was installed. The tips of measuring probes were immersed in the donor chamber and second in the acceptor chamber, which was filled with PBS buffer pH 7.4 [37]. For the experiment, only skin samples of impedance >3 kΩ were applied. These values are similar to the electrical resistance for human skin [38]. After impedance measurement, the donor chamber was removed.

The permeation experiments were done by using Franz diffusion cells (SES GmbH Analyse Systeme, Germany) with diffusion areas of 1 cm^2^. The continuously stirred acceptor fluid was phosphate buffer (pH = 7.4). The content of the acceptor chamber was mixed with a magnetic stirrer. In the acceptor chamber was maintained a constant temperature of 37.0 ± 0.5 °C via thermostat (VEB MLW Prüfgeräte-Werk type of 3280). BC was cut to a 1 cm^2^ size that fitted the surface area of the donor compartment and covered the entire epidermal interface. Next, each membrane was weighed on an analytical balance. At the start of the experiment 30 µL of PBS was applied to the BC. The penetration experiments were performed under occluded conditions by sealing the donor compartment with microscope coverslips [2,11].

The experiment was carried for 24 h. The samples were reported after 0.5 h, 1 h, 2 h, 3 h, 4 h, 5 h, 8 h, and 24 h of stirring. After this time aliquots of the acceptor fluid (0.3 mL) were withdrawn and refilled with fresh buffer at the same pH. The drug concentrations in the acceptor phase were measured by HPLC. After 24 h the BC and skin samples were removed from the Franz diffusion cell. The skin samples were carefully rinsed in PBS solution at 7.4 pH and dried at room temperature. The skin samples were weighed, cut by the diffusion area (1cm^2^), and minced using scissors. Next, skin samples were placed in 2 mL methanol and were incubated for 24 h at 4 °C. After this time skin samples were homogenized for 3 min using a homogenizer (IKA^®^T18 digital ULTRA TURRAX (Staufen im Breisgau, Germany)). The homogenate was centrifuged at 3500 rpm for 5 min. The supernatant was collected and analyzed using HPLC. The cumulative mass (μg·cm^−2^) was calculated based on this concentration. The flux (in μg·cm^−2^·h^−1^) of the ibuprofen and its derivatives through the pigskin into acceptor fluid was determined as the slope of the plot of cumulative mass in the acceptor fluid versus time.

### 3.6. Statistical Analysis

In current studies, all experiments were triplicated and each run was measured in three technical repetitions. Mean values and standard deviations were then calculated by Statistica 13.3 (Statsoft, Tulsa, OK, USA). Prior to analyse difference between tested groups a one-way ANOVA significance test was performed using Statistica 13.3 (Statsoft, USA), comparing only two groups each time.

## 4. Conclusions

The obtained results for bacterial cellulose membranes containing ibuprofen and salts of amino acid isopropyl esters (L-valine and L-leucine) were analyzed to clarify the therapeutic feasibility of BC membranes in transdermal drug delivery. The BC, BC-IBU, BC-[ValOiPr][IBU], and BC-[LeuOiPr][IBU] membranes were obtained simply and effectively. The permeation rate of the compound across BC membranes was the highest for BC-[ValOiPr][IBU] and comparable for BC-[LeuOiPr][IBU] and BC-IBU. The results indicate that by appropriate modification of the active compound it is possible to control the skin permeability. In summary, the obtained membranes based on bacterial cellulose can be used for the transdermal delivery of drugs, including ibuprofen, and their application will be easy and, drug preparation is simple in the form of a single-layer structure. This technology can be successfully used to develop a medical patch.

## Figures and Tables

**Table 1:** Results of mechanical tensile assays obtained BC membranes expressed as Young modulus, elongation at break, and tensile strength. Mean values ± standard deviation, *n* = 5.

Sample	Young Modulus [MPa]	Elongation at Break [%]	Tensile Strength [MPa]
BC	13,807.88 ± 596.43	0.85 ± 0.34	115.53 ± 15.28
BC-IBU	15,304.55 ± 457.68	1.40 ± 0.66	144.32 ± 15.66
BC-[ValOiPr][IBU]	14,958.44 ± 463.33	1.20 ± 0.22	120.67 ± 10.21
BC-[LeuOiPr][IBU]	15,332.48 ± 300.02	1.37 ± 0.06	172.67 ± 12.10

**Table 2:** Average cumulated mass after 24 h permeation of IBU, [ValOiPr][IBU], and [LeuOiPr][IBU] permeated from bacterial cellulose into acceptor fluid pH 7.4 Mean values ± standard deviation, *n* = 3.

Sample	Cumulative Mass (µg IBU cm^−2^)	Drug Permeated 24 h [% Applied Dose]	Skin Accumulation (µg IBU g^−1^)
BC-IBU	87.614 ± 3.737	13.00	293.556 ± 53.197
BC-[ValOiPr][IBU]	104.402 ± 7.875 *	19.49	106.516 ± 16.338 *
BC-[LeuOiPr][IBU]	84.944 ± 5.394	16.89	119.247 ± 13.608 *

* Value is different significantly from control (IBU) (*p* < 0.0001).

**Table 3:** Results for ibuprofen percutaneous permeation. Mean values ± standard deviation, *n* = 3.

Sample	J_SS_ [μg IBU cm^–2^ h^–1^]	K_P_ 10^−7^ [cm^–2^ h^–1^]	L_T_ [min]
BC-IBU	13.16 ± 0.57	2.63 ± 0.11	1.10 ± 0.24
BC-[ValOiPr][IBU]	16.31 ± 0.55	3.26 ± 0.11	4.03 ± 0.36
BC-[LeuOiPr][IBU]	13.22 ± 0.30	2.64 ± 0.06	7.61 ± 0.51

J_SS_—steady-state flux; K_P_—permeability coefficient; L_T_—lag time.

## Data Availability

The data presented in this study are available on request from the corresponding author.

## Supplementary Materials

The following are available online at https://www.mdpi.com/article/10.3390/ijms22126252/s1.
