# Supplementary material for: Transdermal Delivery Systems for Ibuprofen and Ibuprofen Modified with Amino Acids Alkyl Esters Based on Bacterial Cellulose
Source: Int J Mol Sci. 2021 Jun 10;22(12):6252. doi: 10.3390/ijms22126252 (PMC8229522; doi:10.3390/ijms22126252)
Supplement: Supplementary file 1 [file ijms-22-06252-s001.zip › ijms-1252545-supplementary.pdf]

International Journal of *Molecular Sciences*

MDPI

Supplementary Materials

Transdermal Delivery Systems for Ibuprofen and Ibuprofen Modified with Amino Acids Alkyl Esters Based on Bacterial Cellulose

Paula Ossowicz-Rupniewska [1,*], Rafał Rakoczy [2], Anna Nowak [3], Maciej Konopacki [2], Joanna Klebeko [1], Ewelina Świątek [1], Ewa Janus [1], Wiktoria Duchnik [3], Karolina Wenelska [4], Łukasz Kucharski [3] and Adam Klimowicz [3]

1 Department of Chemical Organic Technology and Polymeric Materials, Faculty of Chemical Technology and Engineering, West Pomeranian University of Technology in Szczecin, Piastów Ave. 42, 71-065 Szczecin, Poland; joanna.klebeko@gmail.com (J.K.); ewelinaswiatek94@gmail.com (E.Ś.); ejanus@zut.edu.pl (E.J.)

2 Department of Chemical and Process Engineering, Faculty of Chemical Technology and Engineering, West Pomeranian University of Technology in Szczecin, Piastów Ave. 42, 71-065 Szczecin, Poland; rafal.rakoczy@zut.edu.pl (R.R.); maciej.konopacki@zut.edu.pl (M.K.)

3 Department of Cosmetic and Pharmaceutical Chemistry, Pomeranian Medical University in Szczecin, Powstańców Wielkopolskich Ave. 72, 70-111 Szczecin, Poland; anowak@pum.edu.pl (A.N.); wiktoria.duchnik@pum.edu.pl (W.D.); lukasz.kucharski@pum.edu.pl (Ł.K.); adam.klimowicz@pum.edu.pl (A.K.)

4 Department of Nanomaterials Physicochemistry, Faculty of Chemical Technology and Engineering, West Pomeranian University of Technology in Szczecin, Piastów Ave. 45, 70-311 Szczecin, Poland; karolina.wenelska@zut.edu.pl

* Correspondence: possowicz@zut.edu.pl; Tel.: +48-914494801

Citation: Ossowicz-Rupniewska, P.; Rakoczy, R.; Nowak, A.; Konopacki, M.; Klebeko, J.; Świątek, E.; Janus, E.; Duchnik, W.; Wenelska, K.; Kucharski, Ł.; et al. Transdermal Delivery Systems for Ibuprofen and Ibuprofen Modified with Amino Acids Alkyl Esters Based on Bacterial Cellulose. *Int. J. Mol. Sci.* **2021**, *22*, 6252. https://doi.org/10.3390/ijms22126252

Academic Editor: Jeannine M. Coburn

Received: 24 May 2021
Accepted: 9 June 2021
Published: 10 June 2021

Publisher's Note: MDPI stays neutral with regard to jurisdictional claims in published maps and institutional affiliations.

Copyright: © 2021 by the authors. Licensee MDPI, Basel, Switzerland. This article is an open access article distributed under the terms and conditions of the Creative Commons Attribution (CC BY) license (http://creativecommons.org/licenses/by/4.0/).

Table S1. Ibuprofen content in each BC-membranes.

Sample	Ibuprofen content	
	mg compound/ g membrane	mg active/ g membrane
BC	-	-
BC-IBU	50.0	50.0
BC-[ValOiPr][IBU]	88.6	50.0
BC-[LeuOiPr[IBU]	91.8	50.0

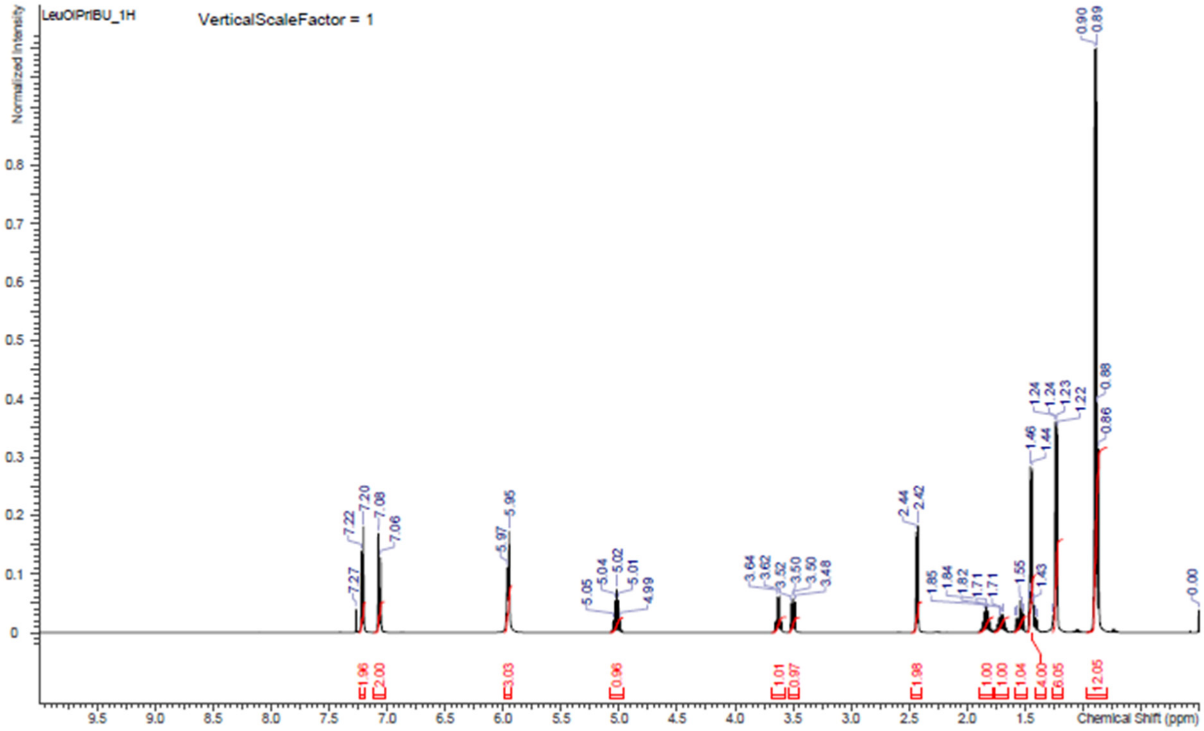

Figure S1. ^{1}H NMR spectra of L-leucine isopropyl ester ibuprofenate.

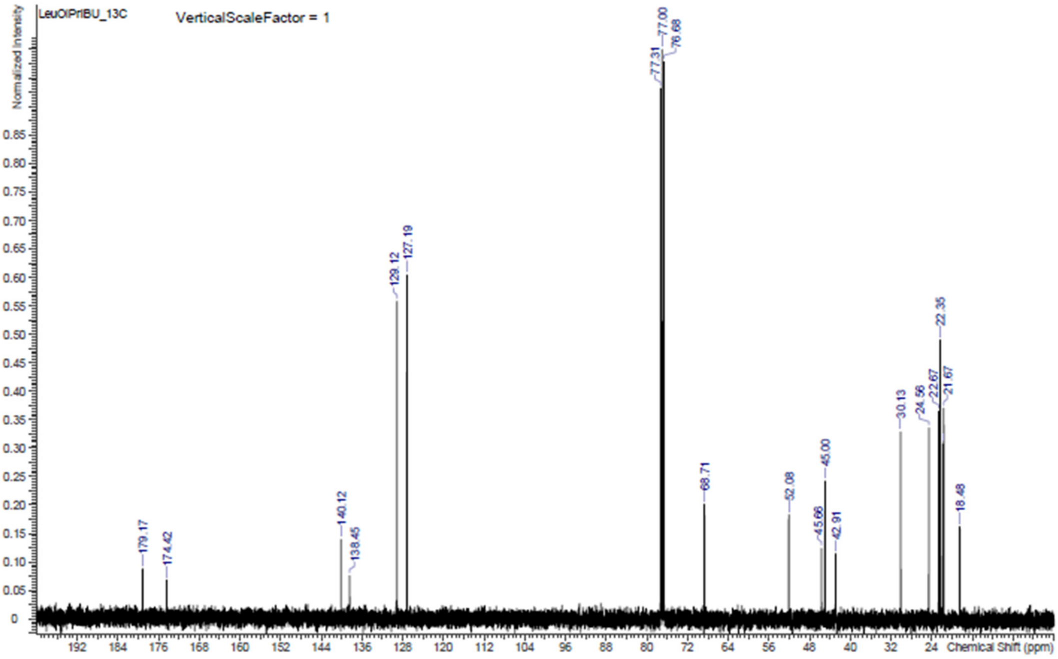

Figure S2. ^{13}C NMR spectra of L-leucine isopropyl ester ibuprofenate.

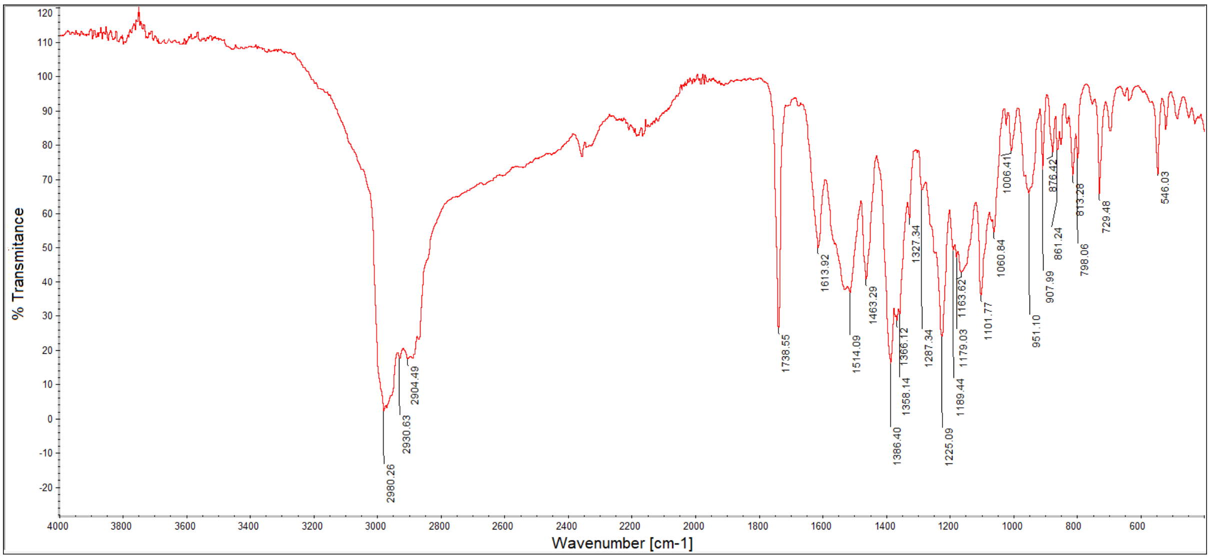

Figure S3. FTIR spectra of L-leucine isopropyl ester ibuprofenate

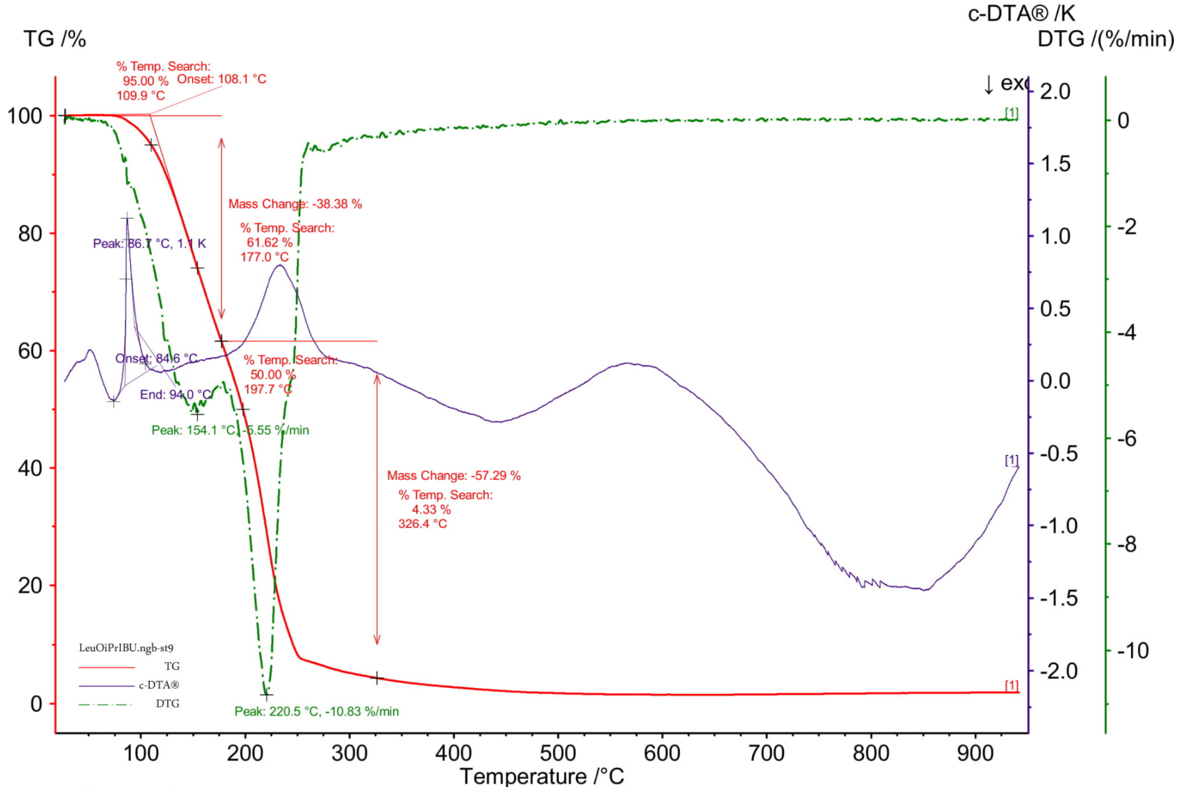

Figure S4. The TG, DTG, and c-DTA curves of L-leucine isopropyl ester ibuprofenate.

Table S2. Statistical differences regarding the cumulative mass of ibuprofen derivatives compared to the control (IBU), evaluated by the Wilcoxon test.

Sample	[ValOiPr][IBU]	[LeuOiPr][IBU]
BC-IBU	p=0.0117* (Z= 2.5205)	p=0.6774 (Z= 0.4200)

* Value is higher significantly from control (*rac*-IBU) (P < 0.0500)

Significantly greater penetration of [ValOiPr] [IBU] was also confirmed by the cluster analysis test - [ValOiPr] [IBU] is far from rac-IBU and [LeuOiPr] [IBU], which form one cluster (Fig. S5). On the other hand, in the case of the

accumulation of the tested compound in the skin, both derivatives form a separate cluster compared to rac-IBU, which confirms that their accumulation is much lower (Fig. S6).

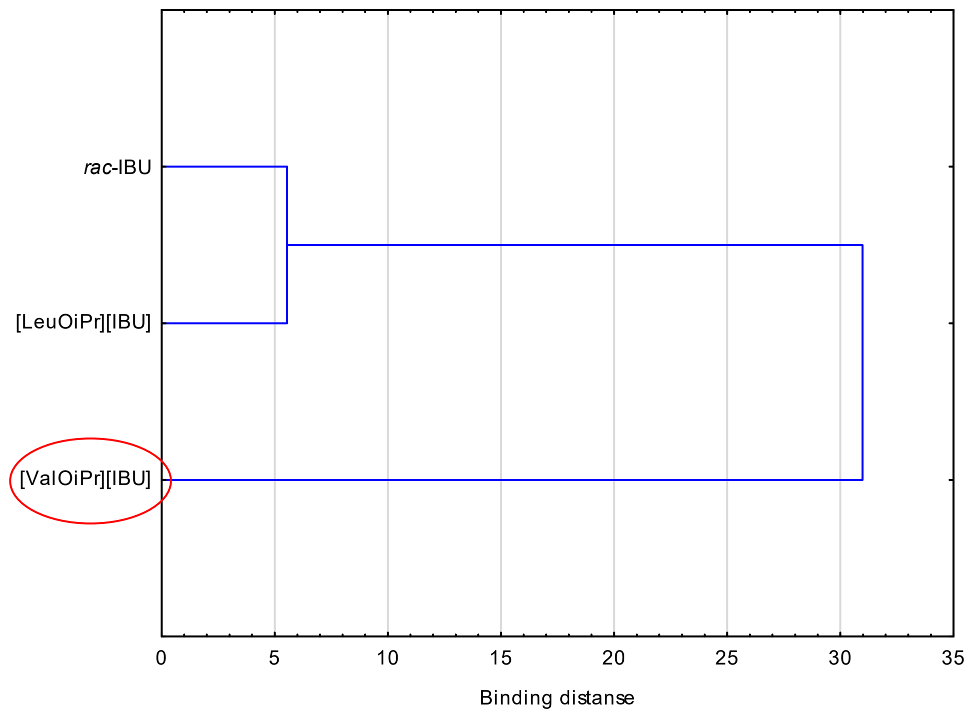

Figure S5. Hierarchical dendrogram of mean amount of IBU, [ValOiPr][IBU], and [LeuOiPr][IBU] cumulated in pigskin after 24 hours penetration.

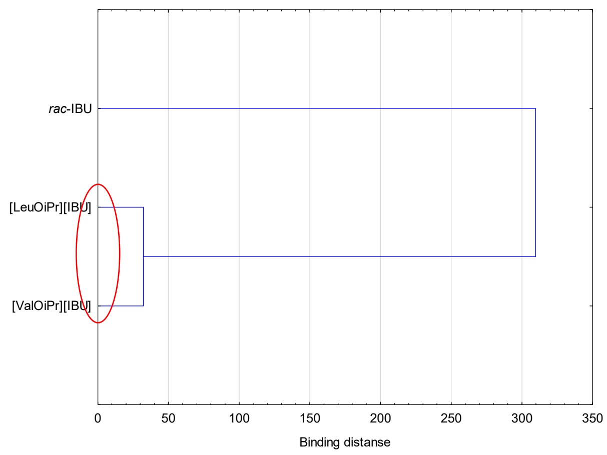

Figure S6. Hierarchical dendrogram of mean cumulated in the skin of IBU, [ValOiPr][IBU], and [LeuOiPr][IBU] after 24 hours penetration.

The mean value of the cumulative mass measured after 24 hours of penetration for [ValOiPr] [IBU] was significantly higher compared to the rest of the compounds as shown in the box plot (Fig. S7). However, in the case of the average cumulative mass calculated from the entire course of penetration (0.5 - 24.0 hours), the values for all compounds are comparable (Fig. S8).

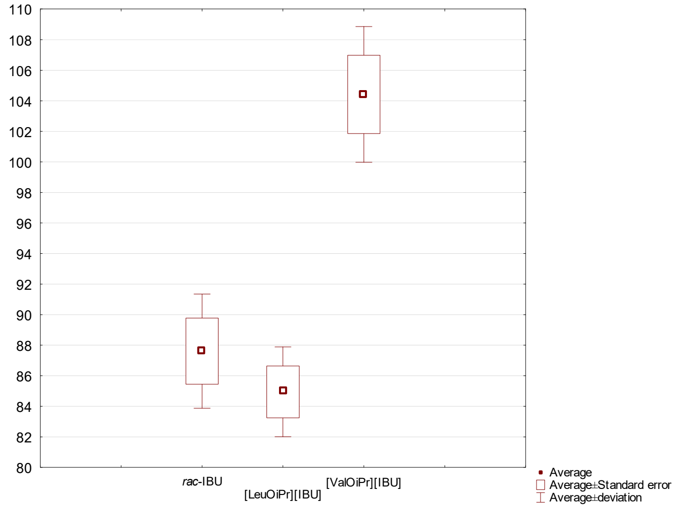

Figure S7. Box and whisker plot of data from a mean cumulative mass of [IBU], [ValOiPr][IBU], and [LeuOiPr][IBU] after 24 hours penetration.

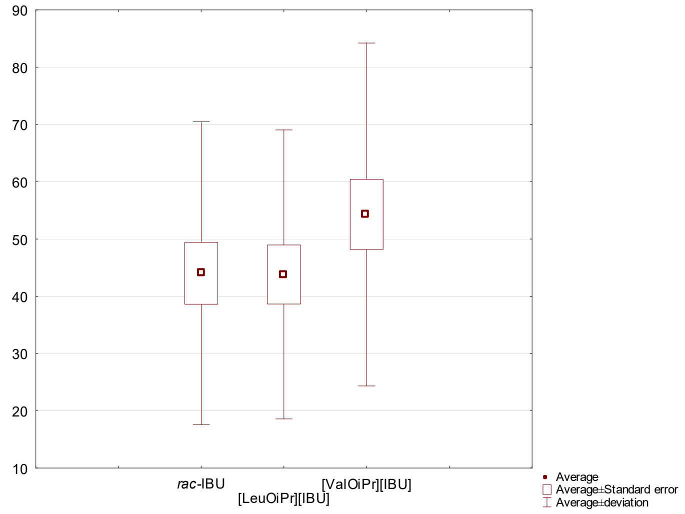

Figure S8. Box and whisker plot of data from a mean cumulative mass of IBU, [LeuOiPr][IBU] and [ValOiPr][IBU] for the entire penetration study (0.5 - 24.0 hours).
